# High-income ZIP codes in New York City demonstrate higher case rates during off-peak COVID-19 waves

**DOI:** 10.3389/fpubh.2024.1384156

**Published:** 2024-06-20

**Authors:** Steven T. L. Tung, Mosammat M. Perveen, Kirsten N. Wohlars, Robert A. Promisloff, Mary F. Lee-Wong, Anthony M. Szema

**Affiliations:** ^1^Noorda College of Osteopathic Medicine, Provo, UT, United States; ^2^Department of Physiology and Biophysics, Renaissance School of Medicine at Stony Brook University, Stony Brook, NY, United States; ^3^Kentucky College of Osteopathic Medicine, Pikeville, KY, United States; ^4^Donald and Barbara Zucker School of Medicine at Hofstra/Northwell, Hempstead, NY, United States; ^5^Three Village Allergy & Asthma PLLC, South Setauket, NY, United States; ^6^Drexel University College of Medicine, Philadelphia, PA, United States; ^7^Icahn School of Medicine at Mount Sinai, New York, NY, United States; ^8^Maimonides Medical Center, Brooklyn, NY, United States; ^9^Division of Pulmonary and Critical Care, Department of Medicine, Donald and Barbara Zucker School of Medicine at Hofstra/Northwell, Hempstead, NY, United States; ^10^Division of Allergy and Immunology, Department of Medicine, Donald and Barbara Zucker School of Medicine at Hofstra/Northwell, Hempstead, NY, United States; ^11^Department of Occupational Medicine, Epidemiology, and Prevention, International Center of Excellence in Deployment Health and Medical Geosciences, Donald and Barbara Zucker School of Medicine at Hofstra/Northwell, Hempstead, NY, United States; ^12^Department of Technology and Society, College of Engineering and Applied Sciences, Stony Brook University, Stony Brook, NY, United States

**Keywords:** COVID-19 pandemic, containment measures, noncompliance, New York City, socioeconomic status, low-income, high-income

## Abstract

**Introduction:**

Our study explores how New York City (NYC) communities of various socioeconomic strata were uniquely impacted by the COVID-19 pandemic.

**Methods:**

New York City ZIP codes were stratified into three bins by median income: high-income, middle-income, and low-income. Case, hospitalization, and death rates obtained from NYCHealth were compared for the period between March 2020 and April 2022.

**Results:**

COVID-19 transmission rates among high-income populations during off-peak waves were higher than transmission rates among low-income populations. Hospitalization rates among low-income populations were higher during off-peak waves despite a lower transmission rate. Death rates during both off-peak and peak waves were higher for low-income ZIP codes.

**Discussion:**

This study presents evidence that while high-income areas had higher transmission rates during off-peak periods, low-income areas suffered greater adverse outcomes in terms of hospitalization and death rates. The importance of this study is that it focuses on the social inequalities that were amplified by the pandemic.

## 1 Introduction

The adverse effects of the COVID-19 pandemic have disparately affected New York City populations, producing considerable variability in the experiences of different socioeconomic strata (1). Polarity between high-income and low-income residential areas in New York City in terms of health outcomes from COVID-19 is still of concern. While other investigators have noted higher mortality rates in low-income neighborhoods, this study delves further into the nuances and patterns of transmission, hospitalization, and death rates during peak and off-peak COVID-19 waves (1, 2). In particular, this study examines the effects of varying levels of compliance with public containment policies and the subsequent health outcomes. In New York State, a total of 77,157 deaths were recorded by March 2023 (3). Although the pandemic affected the entirety of New York State, the bulk of clinical characteristics are concentrated in New York City (2). Pandemic-driven economic downfalls have exacerbated the already existing healthcare inequity gap affecting the low-income communities in large metropolitan areas, such as New York City (1, 2, 4).

Public health data demonstrated that New York City neighborhoods with median incomes below the federal poverty level took the brunt of the pandemic in terms of mortality and transmission rates (4–7). A study conducted regarding social distancing practices and transmission rates among residents of different income levels indicated that low-income individuals had higher infectivity rates due to failed mitigation methods (8). This study supports the belief that economically disadvantaged communities, in which the majority hold blue-collar jobs, have higher rates of COVID-19 transmission throughout the pandemic (2, 4, 5, 9). Previous research demonstrated that non-pharmaceutical interventions, such as social distancing, quantifiably lessen the incidence of infection (6, 7). Such interventions failed to protect low-income individuals whose employment conditions prevented the implementation of the recommended protected measures (6). However, these data do not differentiate rates of transmission during peak and off-peak periods across socioeconomic strata. The responses to COVID-19 mitigation policies among those residents with white-collar jobs, higher degrees of educational attainment, socioeconomic status, and better access to quality healthcare differ from low-income individuals. These factors, in turn, affect their transmission rates during peak and off-peak waves (5, 9–11). Therefore, subtle distinctions in research methodology may have failed to identify important differences in COVID-19 transmission between high and low-income areas. Additional research with improved methodology is required to accept or reject the notion that low-income neighborhoods have higher transmission rates than high-income neighborhoods throughout the entirety of the pandemic (4, 5, 9, 12). This study comprises a diverse population to understand how responses to government policies affect health outcomes in varying socioeconomic groups.

## 2 Methods

United States’ ZIP codes are five-to-nine-digit numeric identifiers used by the Postal Service for mail distribution. They outline specific regions within the United States. In our study, median income of neighborhoods, as defined by ZIP codes, are used as a proxy for socioeconomic status. While median income will vary within a ZIP code, the law of large numbers dictates that the varying median income will coalesce into one of the three enumerated strata provided in this study.

One hundred seventy-seven New York City ZIP codes were used for the analysis of this research. The case rates, hospitalization rates, and death rates data were obtained via NYCHealth’s publicly available data and were extracted on May 5, 2022 (13). Case rate data were defined as cases per 100,000 people based on the date of diagnosis. Weekly case rate data were collected for the timeframe between August 8, 2020, and April 23, 2022, with the week beginning on Sunday and ending on the following Saturday. To keep data consistent with the hospitalization and death rates, the weekly data were allocated into months for comparative analysis. The first day of the month for the case rate was the first Sunday and ended on the Saturday preceding the first Sunday of the next month.

**Figure 1:**
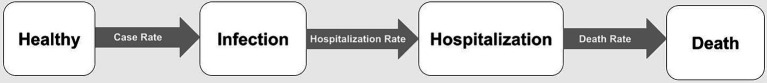
Schematic of disease progression and outcomes, from healthy to death, in a unilateral direction. **Reinfection rate could not be established based on these data.

The hospitalization and death rate data were defined as admissions per 100,000 people based on the date of admission and deaths per 100,000 people based on the date of death, respectively. These rates were collected for months between March 2020 and March 2022 and reported on a monthly basis, with the month beginning on the first day of the month and ending on the last day of the month. The disease progression and outcome schematic demonstrating the association between these metrics is represented in Figure 1.

**Figure 2:**
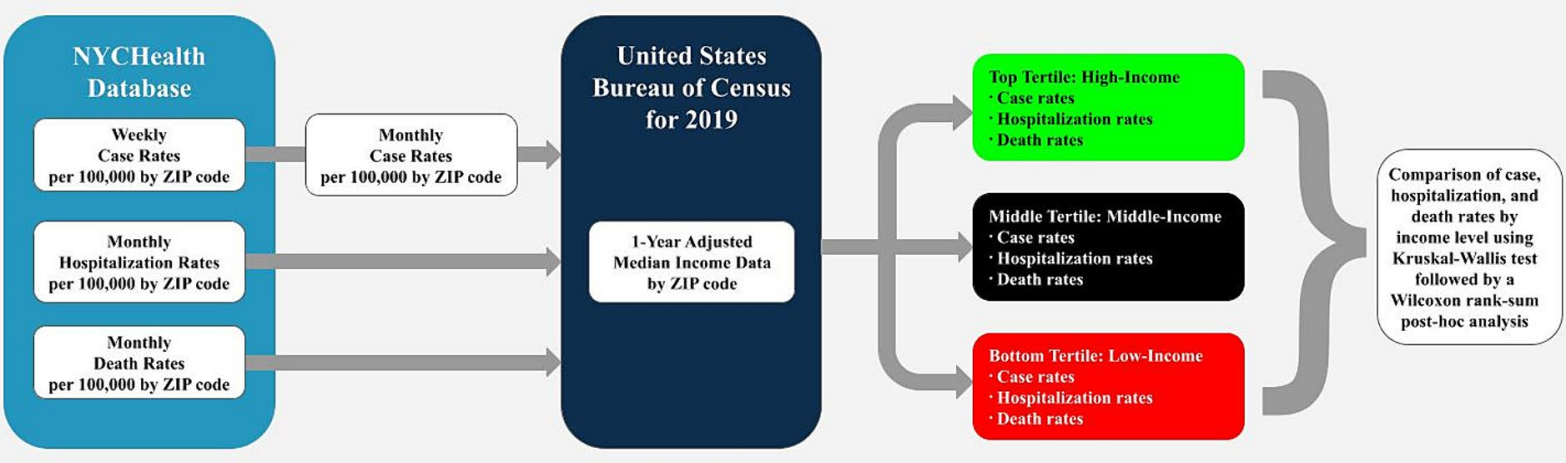
Workflow of data collection, transmission, and analysis. Raw data were collected from NYCHealth’s database and aggregated into monthly data for comparison. Data were parsed into high, middle, and low-income groups using the United States Bureau of Census median income data, then compared using a Kruskal-Wallis nonparametric ANOVA. *Post-hoc* analysis was performed using Wilcoxon rank-sum.

This study utilized the 1-Year Adjusted Median Income Data from the United States Bureau of Census for 2019 (14). In our study, median income data was sorted and parsed into high, middle, and low-income by ZIP code. Each group consisted of 59 ZIP codes. They were placed into these groups based on their order of ascending median total household income. The data collection, transformation, and analysis workflow are displayed in Figure 2. The following ZIP codes: 11001 (Floral Park), 11005 (North New Hyde Park), 11040 (New Hyde Park), and 11096 (Inwood), were not used from the Census data as they were not tabulated into NYCHealth’s data. This is likely due to these regions being counted under adjacent counties.

Peak and off-peak period trends in this study were compared and superimposed with the “Long-Term Trends” case rate report provided by NYCHealth (13). Peak periods occurred from March 2020 to May 2020, November 2020 to April 2021, and December 2021 to February 2022; other timeframes are considered off-peak.

Data organization and statistical analysis were performed on MATLAB R2020b and run on a 64-bit Windows 10 Home operating system using an Intel Core Processor. Case, hospitalization, and death rates between low, middle, and high-income ZIP codes were compared using nonparametric ANOVA testing via the built-in Kruskal-Wallis test, and their respective post-hoc analyses were performed via built-in Wilcoxon rank-sum. A nonparametric test was chosen due to data limitations, such as missing data and varying sample sizes among ZIP codes. All data were compared against a two-tailed distribution with *α* = 0.05.

## 3 Results

**Figure 3:**
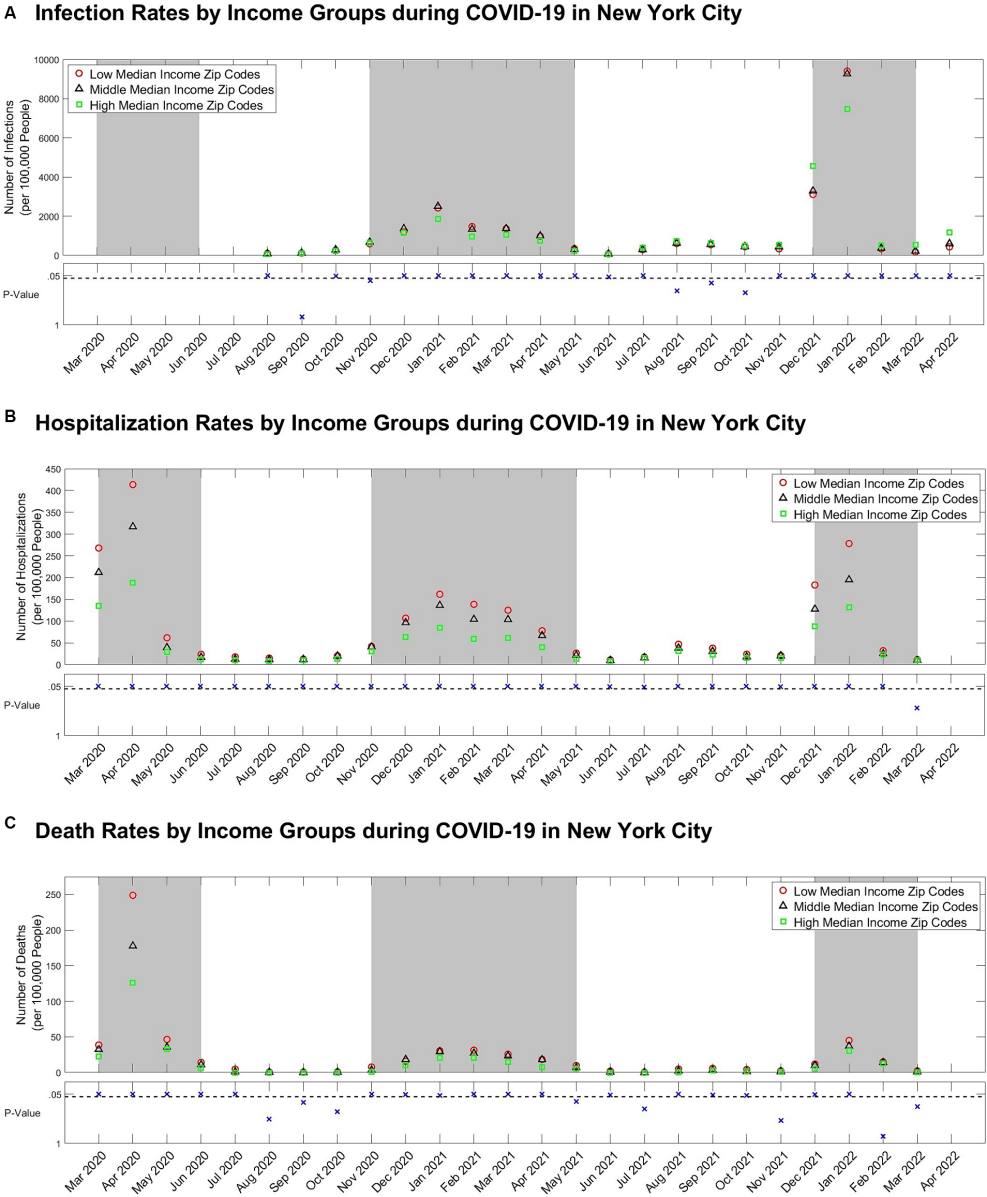
**(A)** Case, **(B)** Hospitalization, and **(C)** death rate per 100,000 people by income-level groups with scatterplots of Kruskal-Wallis *p*-values comparing all three groups below. The dashed line on the scatterplots of *p*-values represents the *α* = 0.05 significance level. The grey backgrounds behind the case, hospitalization, and death rate data indicate peak periods.

Case rates were found to be significantly different among income groups for all months except September and November 2020 and August to October 2021 (*p* < 0.05) (Figure 3A). During peak periods, low-income ZIP codes experienced the highest rate of infection, while high-income ZIP codes experienced the lowest. However, high-income ZIP codes had higher infection rates than their middle and low-income counterparts during off-peak periods.

Hospitalization rates were statistically different among income groups for all months except March 2022 (*p* < 0.05) (Figure 3B). Low-income ZIP codes demonstrated the highest hospitalization rates during peak and off-peak periods, while high-income ZIP codes demonstrated the lowest.

Death rates were statistically different among income groups for all months except August to October 2020, May, July, and November 2021, and February to March 2022 (*p* < 0.05) (Figure 3C). Thus, death rates were highest for low-income ZIP codes during peak and off-peak periods but lowest for high-income ZIP codes.

## 4 Discussion

Hospitalization and death rates were consistently highest among low-income ZIP codes during both off-peak and peak COVID-19 waves. However, these findings are inconsistent with the trends observed in COVID-19 transmission rates. Our findings demonstrate that high-income ZIP codes had the highest case rates during off-peak periods and the lowest transmission rates during peak periods. Importantly, these findings were consistent across multiple peak and off-peak periods. These data support a considerable disparity among the COVID-19 medical experiences within the different socioeconomic strata in New York City.

Behavioral differences may account for the variation in case rates. Possible explanations include increased risky behaviors within high-income communities during off-peak periods, such as leisure travel and high-density gatherings. Conversely, self-imposed isolation, working from home, and fleeing New York City during peak periods may contribute to reduced case rates (15–17). By contrast, low-income communities comprise a greater proportion of essential workers who do not have the ability or financial resources to flee the city or work from home. Additionally, low-income communities face additional risks for transmission, such as documentation status, lack of access to green spaces, and housing density (18–20).

Importantly, New York City enforced mitigation policies, such as remote learning, social distancing, capacity limits, travel restrictions, and business closures, from March 2020 to June 2021 (21–23). The differences in case rates in high-income communities during peak and off-peak periods suggest fluctuating and inconsistent voluntary adherence to preventive policies. Noncompliance by high-income individuals produced a source of contagion counterproductive to government and public health policies intended to slow down the spread of the pandemic.

Our research supports the concept that the effects of noncompliance attributed to the perpetuation and spread of the COVID virus resulted in increased morbidity and mortality. Our findings are consistent with previous research, which documented that noncompliance is more frequent in individuals with higher educational attainment and socioeconomic status (24). Stringent recommendations were advised, but our study demonstrates the effects of deviance from these policies. This represents a failure of government mandates. This lack of compliance increased casualties for all communities but was especially felt by low-income communities, comprised primarily of blue-collar and essential workers. Although COVID sceptics, deniers, and dissidents are condemned for their role in perpetuating the pandemic, high-income individuals who regularly defied government mandates share responsibility for the morbidity and mortality of the pandemic. However, the average incubation period has been shown to be 6.5 days, which may have led to infectious but undetected individuals unconsciously spreading the virus (17, 25).

Increased hospitalization and death rates in low-income neighborhoods during peak and off-peak periods may be the result of decreased access to quality healthcare and underlying comorbidities (7, 17, 25). Prior studies concluded that low-income individuals have more health issues, leading to disproportionately higher harm as a result of COVID-19 (18). Decreased rates of vaccination in low-income communities may also contribute to increased morbidity and mortality (25). Lower numbers of healthcare delivery systems and professionals serving people under the poverty threshold may be an additional factor (1, 4, 5, 9).

This paper provides evidence that low-income communities had the highest rates of hospitalization and death during off-peak periods despite having the lowest number of cases. This demonstrates that the burden of the COVID-19 pandemic was felt most substantially by low-income communities during off-peak periods. Additionally, this finding contradicts previous evidence that low-income communities had higher transmission rates throughout the entire pandemic, as well as the notion that increased morbidity and mortality in low-income communities resulted from inadequate mitigation methods (8). Previous research emphasized that essential workers, comprised largely of blue-collar and low-income individuals, were the main source of contagion throughout the pandemic (2, 4, 5, 9). Contrarily, these findings suggest that noncompliance in high-income communities contributed to the disparities in health outcomes. Therefore, this study accentuates the complex relationship between these socioeconomic groups during the pandemic.

The low granularity of data in this study is a possible limitation. Some ZIP codes have considerable socioeconomic, racial, and ethnic diversity. The groups utilized in this study do not reflect any government or organizational socioeconomic classifications – such as “impoverished,” but rather, the delineation allows for gross stratification of socioeconomic standing. Therefore, the median income may not accurately represent the wealth of its citizens. Additionally, rates were recorded based on the site of diagnosis, death, or hospitalization, not necessarily the patient’s residence, further limiting this study’s findings (13). The low, medium, and high-income designations used in this study are not related to definitions of wealth, such as poverty levels. Moreover, adjustments for confounding factors, such as age, race, underlying health conditions, and access to healthcare services, were not made. Future research should investigate the effect of these factors on the observed socioeconomic inequalities. Lastly, a more in-depth analysis is required to confirm the role of the inferred behavior on health outcomes during the COVID-19 pandemic.

Understanding the effects of non-compliance in high-income communities is essential for developing effective public health policy. Based on our findings, it is likely that behavior in high-income communities increased caseload and contributed to the morbidities and mortalities in low-income neighborhoods. Therefore, it is important that high-income communities avoid risky behaviors such as travel and large social gatherings during public health crises to mitigate the burden of disease experienced by their low-income counterparts. Public policy initiatives should be cautiously cognizant of the spectrum of socioeconomic groups when forging healthcare policy to focus on methodologies that reduce transmission. Future public policy should consider subtle differences in compliance amongst varying socioeconomic groups to attempt to produce equitable health outcomes.

## Data availability statement

The original contributions presented in the study are included in the article/Supplementary material, further inquiries can be directed to the corresponding author/s.

## Author contributions

MP: Conceptualization, Investigation, Methodology, Writing – original draft, Writing – review & editing. ST: Data curation, Formal analysis, Software, Validation, Visualization, Writing – original draft, Writing – review & editing. KW: Validation, Writing – original draft, Writing – review & editing. RP: Supervision, Writing – original draft, Writing – review & editing. ML-W: Supervision, Writing – original draft, Writing – review & editing. AS: Supervision, Writing – original draft, Writing – review & editing.
